# Editorial: Psychedelic substances and neurological diseases: from basics to clinical application

**DOI:** 10.3389/fphar.2026.1816812

**Published:** 2026-03-16

**Authors:** Alessandra Linardi, Ariadiny Lima Caetano, Rodrigo Portes Ureshino

**Affiliations:** 1 Department of Physiological Sciences, Santa Casa de São Paulo School of Medical Sciences, São Paulo, Brazil; 2 Department of Biological Sciences, Federal University of Sao Paulo, São Paulo, Brazil; 3 Laboratory of Molecular and Translational Endocrinology, Escola Paulista de Medicina, Federal University of Sao Paulo, São Paulo, Brazil

**Keywords:** beta-carbolines, drug addiction, nervous system damage, neuroinflammation, phosphoproteomics, psychedelics, psychiatric disorders, social hierarchy

Scientific interest in psychedelic substances has re-emerged, offering new perspectives on the treatment of neuropsychiatric and neurological disorders. Historically, psychedelics have been associated with a wide spectrum of positive and negative effects, encompassing ritualistic and spiritual use, recreation and expansion of consciousness (Aicher et al., 2024). Importantly, psychedelics have been shown to induce neuroplasticity and alleviate symptoms associated with various neurological and psychiatric disorders (Lashgari et al., 2025). Clinical trials and basic research have demonstrated promising results in the use of these substances to treat conditions such as depression, anxiety, post-traumatic stress disorder, and even neurodegenerative diseases such as Alzheimer’s disease, tauopathies and Parkinson’s disease (Davis et al., 2021; Bradley et al., 2025; Cury et al., 2025; Vrechi et al., 2025). This Research Topic aims to provide insights on the fundamental mechanisms of psychedelic action, its historical and contemporary uses, and emerging evidence supporting its effectiveness in clinical settings, with a primary focus on psychoactive substances from the perspective of toxicity and therapeutic potential.

Regarding the mechanisms involved in neurotoxicity and its clinical implications, presented a review of six commonly abused recreational drugs—methamphetamine, cocaine, synthetic cathinones, ketamine, nitrous oxide, and heroin (morphine derivative)—and the neurotoxicity associated with these substances. Although acute morphine withdrawal promotes anxiety-like behavior in mice (Masukawa et al., 2020), Wang et al. highlight that, despite their distinct pharmacokinetic and pharmacodynamic profiles, these substances share convergent neurotoxic mechanism involving oxidative stress, mitochondrial dysfunction, excitotoxicity, and neuroinflammation. This review is particularly relevant because it discusses the relationship between dissociative effects and neuroplasticity in the context of oxidative stress at the cellular level. Similarly, psychedelics such as ketamine and ethanol promote oxidative stress and induce apoptosis in neuronal cell models (Castelhano et al., 2025). Therefore, this review addresses the limitations of current pharmacotherapy, and highlights the need for new therapeutic approaches in cases of intoxication and abuse, including potential targets involved in neuroinflammation and antioxidant agents.

Expanding the discussion beyond molecular toxicity, Xu et al. introduce a critical social dimension to drug reinforcement and vulnerability to addiction. Their original research demonstrates that social hierarchy modulates methamphetamine reinforcement in rats and is associated with distinct phosphoproteomic signatures in the nucleus accumbens. The authors reported higher levels of Histone Deacetylase 4 (HDAC4) phosphorylation in dominant rats, a protein previously described as playing critical roles in drug addiction. Notably, after behavioral training that altered the animals’ social hierarchy between dominant and subordinate rats, the differences in HDAC4 phosphorylation levels were abolished; similarly, methamphetamine reinforcement was reversed. Collectively, these findings demonstrate the critical role of protein phosphorylation in regulating social hierarchy and its impact on drug reinforcement, offering a new pharmacological perspective on the social context of addiction.

Focusing on the neuromodulation of anxiety and depression, the mini-review by Li et al. discusses the emerging role of trace amine–associated receptors (TAARs), particularly TAAR1, as potential therapeutic targets. Preclinical evidence that TAAR1 agonists exert antidepressant and anxiolytic effects in animal models, whereas TAAR5 antagonism reduces anxiety-related behaviors. Previously considered minor neurochemical components due to their low cerebral concentrations, trace amines are now increasingly recognized as key modulators of dopaminergic and serotonergic neurotransmission and stress-related responses. Thus, by contextualizing the role of TAARs in stress-related psychiatric disorders and depression, this mini-review substantially expands the pharmacological landscape of psychedelic science and suggests alternative or complementary targets for future therapeutic strategies.

From a complementary perspective, Bianco et al. integrate traditional psychoactive compounds with contemporary Research Topic related to neuroinflammation, viral infection, and long-term neurological sequelae. This original article investigates the anti-inflammatory and neuroprotective effects of *B. caapi* extract and its β-carbolines, harmine and harmaline, in a neuroinflammation model using SH-SY5Y cells and presents cytokine profiles from COVID-19 patients for clinical context. The study demonstrates that β-carbolines and *B. caapi* extract attenuate LPS-induced production of pro-inflammatory cytokines and modulate NF-κB expression in neuronal cells. Additionally, the modulation of ACE2 and TMPRSS2 expression by *B. caapi* extract and β-carbolines suggests potential relevance for neuro-COVID–related mechanisms.

From an integrative and humanistic perspective, Tunsdad et al. demonstrated the importance of identifying integration, ego dissolution, and emotional overcoming as key predictors of self-reported wellbeing after the use of classical psychedelics, as reported by Norwegian participants recruited online. In this context, factors such as ceremonial settings, contact with nature and therapeutic intent also contributed, although effects of less magnitude. Therefore, considering the broader context of neurological and psychiatric diseases, this work highlights how experiential and phenomenological dimensions can interact with neural plasticity to produce lasting changes in daily life.

Taken together, the articles in this Research Topic illustrate the multidimensional nature of psychedelic and psychoactive substance research. They integrate multiple perspectives, encompassing intracellular signaling, phosphoproteomics, and neuroinflammation, while also addressing social hierarchy and subjective experience. An important conclusion is that neurological outcomes from these studies are influenced by dynamic interactions between biological mechanisms and contextual factors. Neurotoxicity and neuroprotection, addiction and therapeutic benefit, vulnerability and resilience are influenced by social context, environment, and individual neurobiology; collectively, these factors contribute to diagnosis, clinical presentation, and social interactions. Consequently, integrative approaches are needed to connect intracellular and molecular mechanisms, neuroinflammatory processes, and epigenetic regulation to experiential dimensions and social context. As interest in psychedelic-assisted therapies continues to grow, careful attention to safety, context, and individual variability will be fundamental to maximizing therapeutic benefit and minimizing adverse effects. Moreover, pathways linked to neuroplasticity may also contribute to neurotoxicity depending on dose and context, underscoring the need for careful interpretation. Progress in this field will require rigorous controlled trials, standardized protocols, long-term follow-up, and mechanistic studies capable of distinguishing true therapeutic effects from nonspecific or expectancy-driven responses. Therefore, the Research Topic “Psychedelic Substances and Neurological Diseases: From Basics to Clinical Application” offers a multidisciplinary perspective on how psychoactive substances can modulate neurological disorders such as depression and anxiety, as well as induce experiences that can promote complete physical, mental, and social wellbeing.
